# A Standard System to Study Vertebrate Embryos

**DOI:** 10.1371/journal.pone.0005887

**Published:** 2009-06-12

**Authors:** Ingmar Werneburg

**Affiliations:** Paläontologisches Museum und Institut der Universität Zürich, Zürich, Switzerland; Ecole Normale Supérieure de Lyon, France

## Abstract

Staged embryonic series are important as reference for different kinds of biological studies. I summarise problems that occur when using ‘staging tables’ of ‘model organisms’. Investigations of developmental processes in a broad scope of taxa are becoming commonplace. Beginning in the 1990s, methods were developed to quantify and analyse developmental events in a phylogenetic framework. The algorithms associated with these methods are still under development, mainly due to difficulties of using non-independent characters. Nevertheless, the principle of comparing clearly defined newly occurring morphological features in development (events) in quantifying analyses was a key innovation for comparative embryonic research. Up to date no standard was set for how to define such events in a comparative approach. As a case study I compared the external development of 23 land vertebrate species with a focus on turtles, mainly based on reference staging tables. I excluded all the characters that are only identical for a particular species or general features that were only analysed in a few species. Based on these comparisons I defined 104 developmental characters that are common either for all vertebrates (61 characters), gnathostomes (26), tetrapods (3), amniotes (7), or only for sauropsids (7). Characters concern the neural tube, somite, ear, eye, limb, maxillary and mandibular process, pharyngeal arch, eyelid or carapace development. I present an illustrated guide listing all the defined events. This guide can be used for describing developmental series of any vertebrate species or for documenting specimen variability of a particular species. The guide incorporates drawings and photographs as well as consideration of species identifying developmental features such as colouration. The simple character-code of the guide is extendable to further characters pertaining to external and internal morphological, physiological, genetic or molecular development, and also for other vertebrate groups not examined here, such as Chondrichthyes or Actinopterygii. An online database to type in developmental events for different stages and species could be a basis for further studies in comparative embryology. By documenting developmental events with the standard code, sequence heterochrony studies (i.e. Parsimov) and studies on variability can use this broad comparative data set.

## Introduction

*“I will discuss this topic very briefly, because it seems to be redundant to me to describe things with words that everyone realises easily when reckoning the drawings.”* (Richard Semon, 1894c, on the development of the echidna's body shape [1: page 72]).

Documenting embryological development is a particular challenge for comparative anatomy and evolutionary research [2]–[4]. During the last decade the value of developmental characters in a phylogenetic framework was emphasised and new parsimony-based methods were developed to analyse phylogenetic patterns in embryology [5]–[8]. Unfortunately, at present, a common language for defining developmental characters, which may serve as a basis to create a large comparable fundament for comparative embryology research, does not exist.

**Figure 1:**
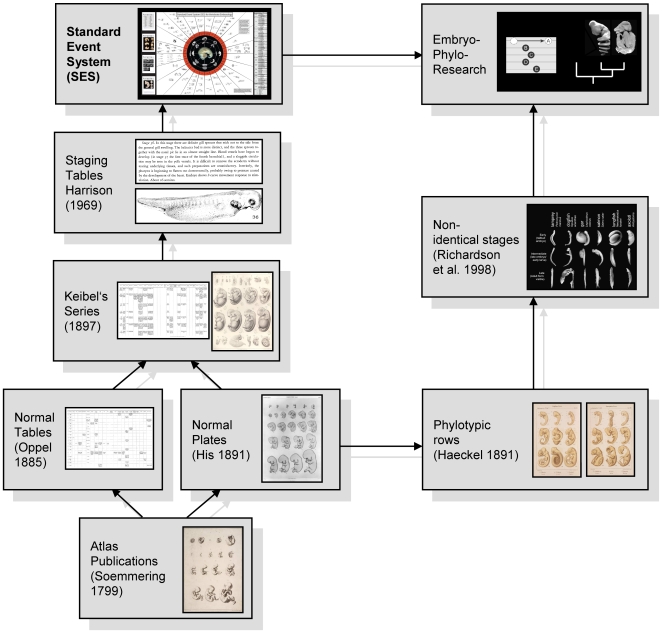
Scheme of the history of documenting embryology and of embryological research. Illustrations modified from cited references and Jeffery et al. [7], for the presented Standard Event System a shortcut of the supplementary poster (Poster S1) is used. For historical details see text and Hopwood [10], [14].

Documentation of embryological development (**Figure 1**) began with early typological *atlas publications* of human embryos such as that of Soemmerring [9]. Herein series of the rare available, aborted embryos were presented, which were ordered chronologically by days and months after the last menstruation cycle of the mother. The authors “sought to see beyond mere individuals to represent types” [10]. Wilhelm His [11] refrained from typology and developed a *normal plate* system (*“Normentafeln”*) where individuals are represented showing a probably ‘normal’, non pathological development. Oppel [12] established extensive embryonic *normal tables*, which document the development of internal organs. Franz Keibel was the first who unified these two approaches (**Figure 1**) and edited a 16 volume series of *Normal Plates of the Development of the Vertebrates* beginning with the ‘normal development’ of the domestic pig [13]. In this large format series, high standard drawings as well as a tabular and written documentation of the developmental processes and variability within one species were presented. Although he praised Keibel's work, Hopwood [10], [14] criticised that the project failed as a whole because no synthesis of embryological patterns and no general conclusion about variability could be elucidated. In the first part of the 20^th^ century Ross G. Harrison designed a set of *stages* for *Amblystoma* based on his survey on several specimens (*staging table*/*normal stages*). He standardised them by a series of drawings and by describing characters typical for each stage in a text format [completely published: 15]. With the rising interest in comparative embryology numerous staging tables in the Harrison-style were published for the main vertebrate groups and established as a “common language” between laboratories. But they were treated more as tools rather than results [i.e. 16, chicken embryology]. During the last decades the use of clade representative ‘model organisms’ was questioned [17]–[19], highlighting problems created by limited sampling and the biases in phylogenetic comparisons in Evo-Devo studies. To circumvent this problem, an increasing number of scientists has attempted to establish new organisms as ‘models’ throughout vertebrates [i.e. 20]–[23].

The recent development of sequence heterochrony (temporal shifts in development) methods [5]–[8], [24]–[27] set the basis to analyse different developmental patterns between species in a phylogenetic context. These methods compare *events* i.e., newly occurring characters in development [28]. Further studies calculate the variation of developmental sequences [29] in a phylogenetic framework [30]. Up to date no comparable standard has been developed to describe and to depict developmental events.

This study samples mostly turtles, but includes characters relevant for vertebrates in general. First descriptions of turtle embryology were given by Agassiz [31], Rathke [32] and Parker [33] in the 19^th^ century. In the second part of the 20^th^ century two studies, following the Harrison-style of staging tables, influenced turtle developmental studies. On the one hand Yntema's [34] study on the ‘model organism’ *Chelydra serpentina* (Cryptodira) set a long lasting 27 staged standard for staging non-marine turtle embryos. Several authors have pointed out differences in development of other species, such as timing in limb development [35]–[37] or distribution of scales and pigmentation patterns [38]. The applicability of described characters of a certain species to those of a related species was questioned [39]. In the last few years the development of external characters was described in detail for several turtle species [38]–[42], but the stages designed by Yntema [34] remained standard. On the other hand, observing six species, Miller [43] proposed a 31 staged standard for marine turtle (Chelonioidea) embryology [44].

When comparing the embryology of diverse vertebrate groups [6], [45] the necessity of a standard to describe developmental features in early development is obvious. In turtles for example, authors have focused on the development of specific elements such as the urogenital system and the head [38] or the limbs [46]. Other authors, who have a different approach, described a few external features superficially [47].

**Figure 2:**
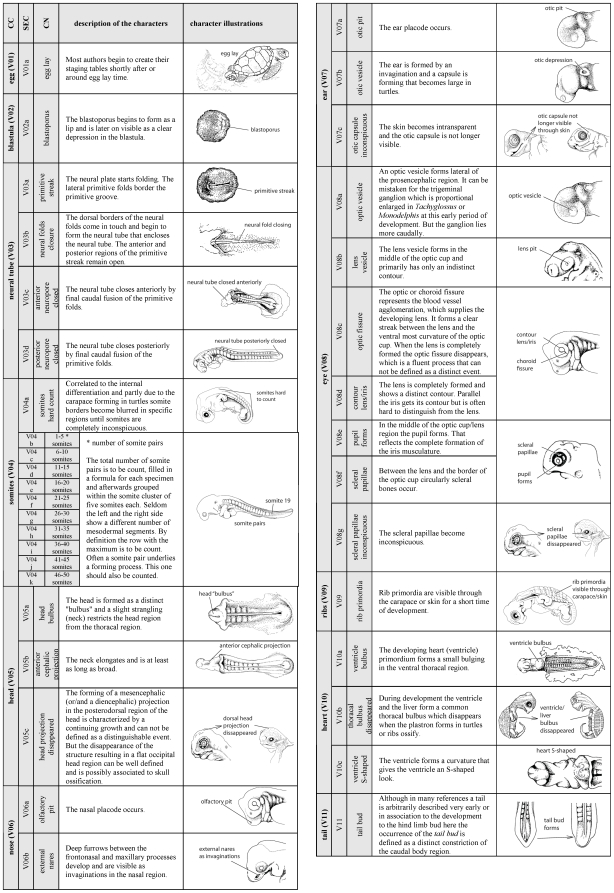
Definition and illustration of external morphological characters that describe a developmental event (Page 1 of 3). Standard codes (V01a etc.) as defined in the text. CC = Character complex, CN = Character name, SEC = Standard Event Code. Character complexes as occur and evolved within V = Vertebrata, G = Gnathostomata, T = Tetrapoda, A = Amniota or only within S = Sauropsida. Illustrations modified after Guyout et al. [40], Renous et al. [44] and Mahmoud et al. [89], with exception from “V01a”, “V13e” and “V14a”, which are from different sources. Except for few obvious drawings: left = anterior, right = posterior. Nomenclature follows mainly Schoenwolf [90]. Please note the pictures are only used for character illustration and do not necessarily reflect the first occurrence of the character in the shown species. For continuation compare Figure 3–4.

A new method to comprehensively describe developmental characters throughout all vertebrate groups is presented. Therefore I elaborated a detailed description of 104 developmental characters of external morphology (**Figure 2** **–** [Fig pone-0005887-g003] **4**). Based on these descriptions an extendable type-in-formula is provided to document character sets of diverse species. The problems encountering when ‘staging’ developmental series, and a solution to them, are discussed. Also ideas to establish an online database are presented, in which inter- as well as intraspecific variability can be documented. Here I follow the approach of Oppel [12] who documented intraspecific variation in his extended normal tables.

**Figure 3 pone-0005887-g003:**
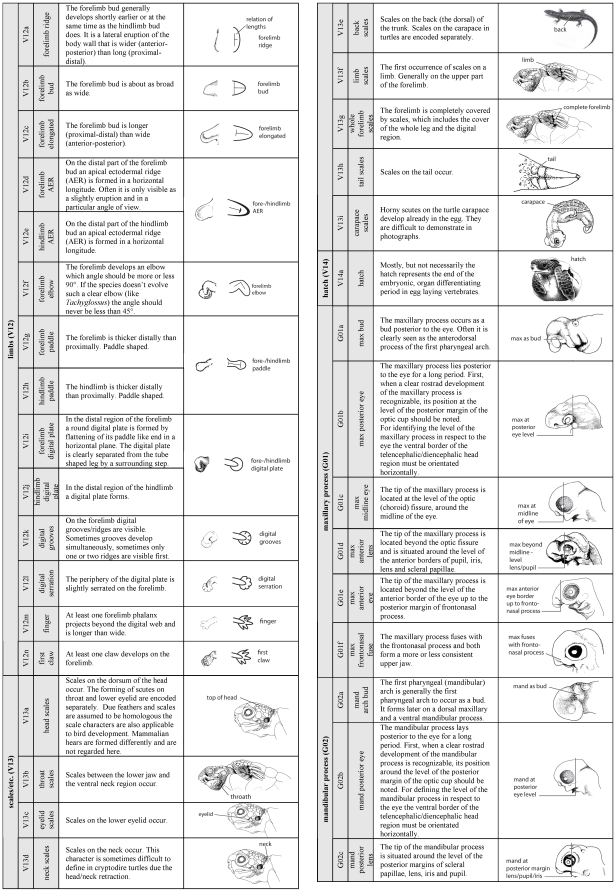
Character definition and illustration (Page 2 of 3). For description and continuation of the list compare Figure 2 and 4.

**Figure 4:**
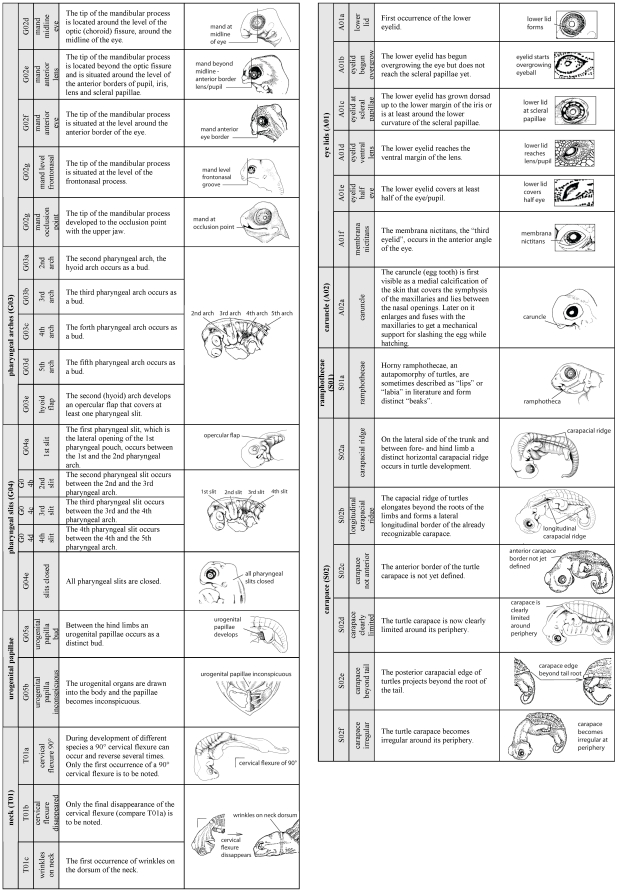
Character definition and illustration (Page 3 of 3). For description and continuation of the list compare Figure 2–3.

## Results

**Figure 5:**
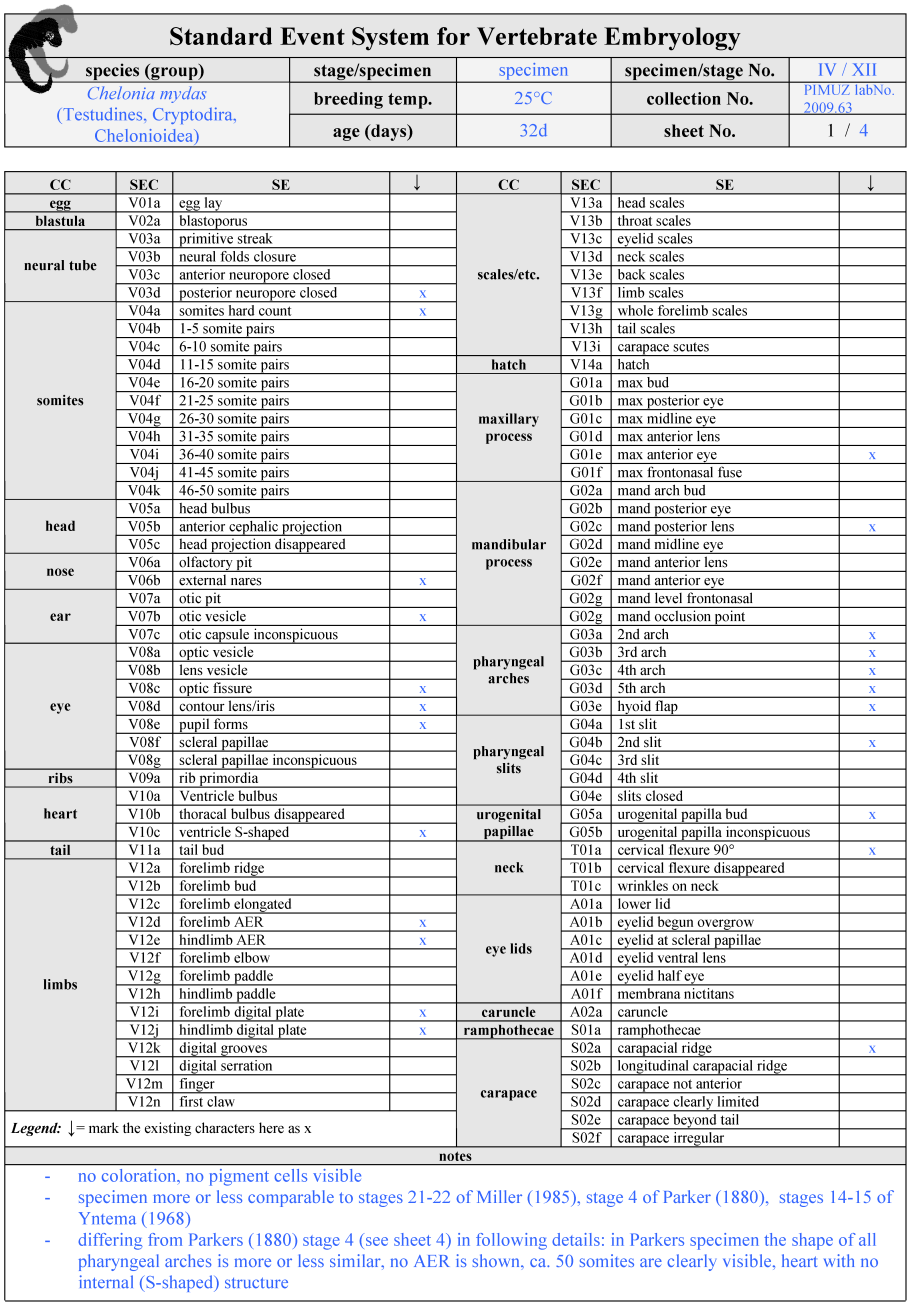
Initial page of a filled SES-formula. A 32 day old *Chelonia mydas* (Green sea turtle) embryo is used as a case example to illustrate how to fill an SES-formula. Embryological characters as described in Figure 2–4 are listed in a check list format. Below additional space is offered for further observations like on proportion or colouration. Each sheet of the SES-formula (see also Figure 5–7) has the same head listing species name, breeding temperature, embryo age, catalogue number, as well as one field to type in if either the formula is used to describe one single specimen or one stage (synopsis of several more or less similar specimens). For further instructions how to use the formula see Discussion.

I introduce a *Standard Event System* (SES) to document embryological development comparatively. Using this term I avoid typological terms such as staging table, normal stage or normal development. Embryonic series are to be arranged in defined SES-stages. These SES-stages are described and illustrated in a SES-formula (**Figure 5** **–** [Fig pone-0005887-g006] [Fig pone-0005887-g007] **8**, **Table S1** **,** **S2**). The SES-formula can be used either to describe only one specimen or to define features that characterise the synopsis of several specimens representing one single - author defined - stage. In the formula I also offer space for a traceable cataloging, for additional descriptions of specimen/species identifying characters such as pigmentation, carapace shape or scute/feather-arrangement features, as well as space for drawings and photographs. This formula offers a check-list for SES-characters (**Figure 5**) presented in a particular specimen/stage. Each SES-character is simply encoded by a SES-code comprising 104 characters thoroughly described in **Figure 2** **–** [Fig pone-0005887-g003] **4**. The three-part SES-code is generated for characters that evolved and differentiated within Vertebrata (V), Gnathostomata (G), Tetrapoda (T), Amniota (A) or only within Sauropsida (S). Character complexes are listed such as the “maxillary process (G01)” of Gnathostomata or “eye lids (A01)” of Amniota. For each event that occurs within the referred character complex, a small letter is used: i.e. “maxillary process present as a bud (G01a)”, “maxillary process fuses with frontonasal process (G01f)” or “lower eyelid covers half of the eye (A01e)”. Using this scheme the table is extendable. For example, including more Mammalia (M) species into the study, new character complexes can be comparatively added, such as “birth (M01a)”, “hair on the top of the head (M02a)” or “hair on the throat (M02b)”. In this way, beside external morphological characters also internal morphological, genetic, physiological and molecular characters can be included easily. For convenience, a printable formula template (**Table S1** **,** **S2**), one example of using such a formula (**Figure 5** **–** [Fig pone-0005887-g006] [Fig pone-0005887-g007] **8**), and a template for a printable laboratory poster depicting all SES-characters (**Figure 9**, **Poster S1**) are provided.

**Figure 6 pone-0005887-g006:**
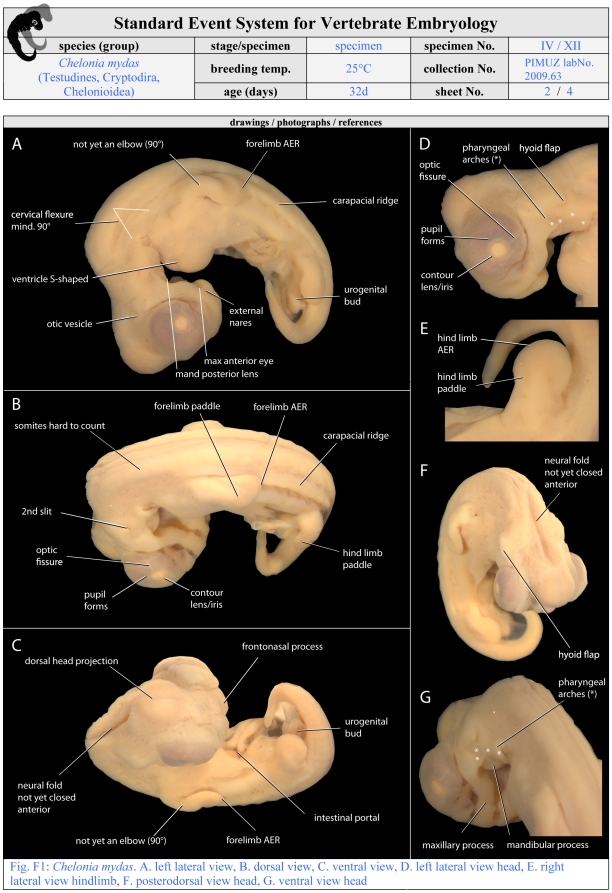
Second required page of a filled SES-formula. On this page all observed SES-characters are depicted on illustrations of the specimen described. For comparability photographs should be made using a light microscope. A lateral, dorsal and ventral view of the whole body is required and more detailed illustrations are optional. Additional pages of this kind are imaginable. For further details see Figure 5 and Discussion.

**Figure 7 pone-0005887-g007:**
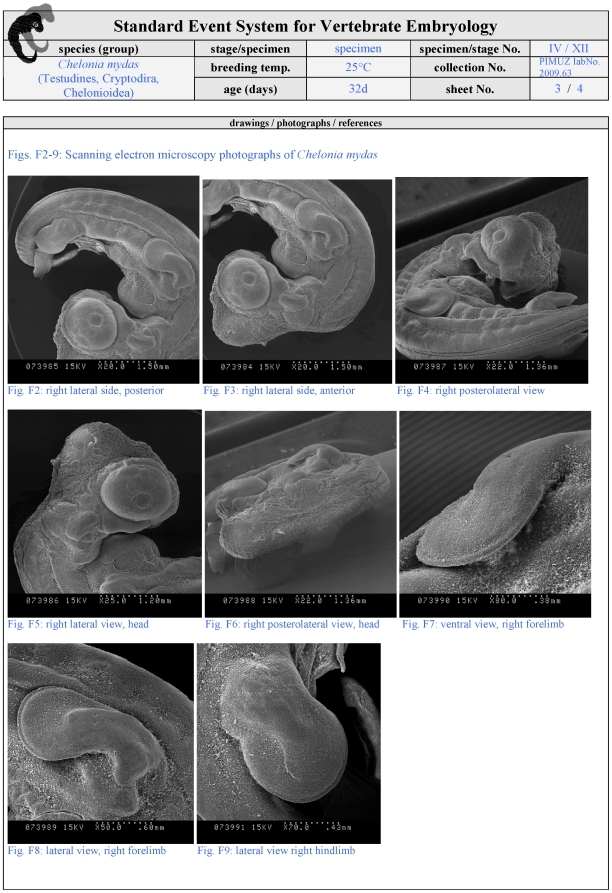
First optional page of a filled SES-formula. On this page additional illustrations may be provided that are made using non-light-microscopy-observations like scanning electron microscopy. These pictures should not be used to illustrate SES-characters that are not visible in light microscopy. For further details see Figure 5 and Discussion.

**Figure 8:**
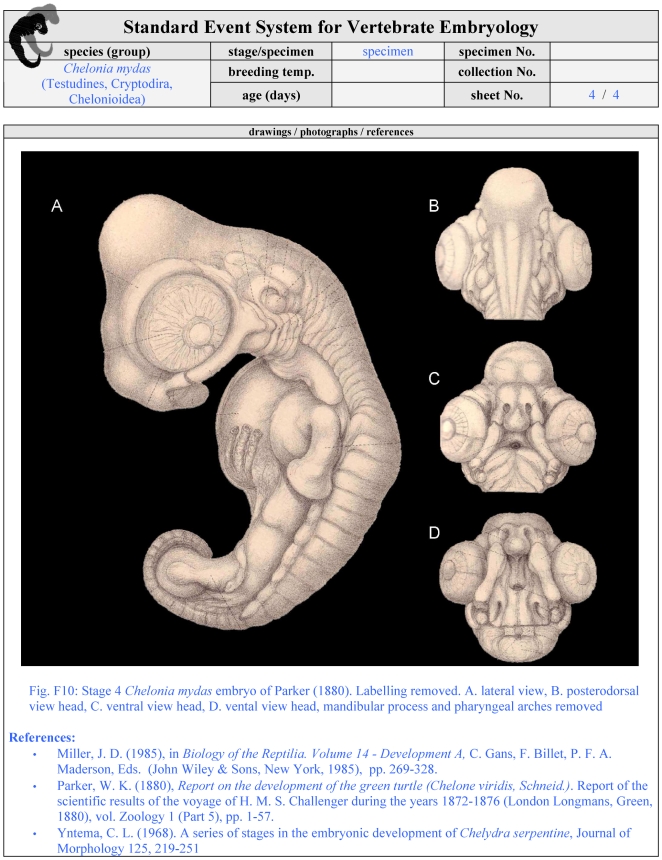
Second optional page of a filled SES-formula. On this page illustrations of reference papers may be provided showing drawings/photographs of similar specimens/stages as the described one. For further details see Figure 5 and Discussion.

**Figure 9:**
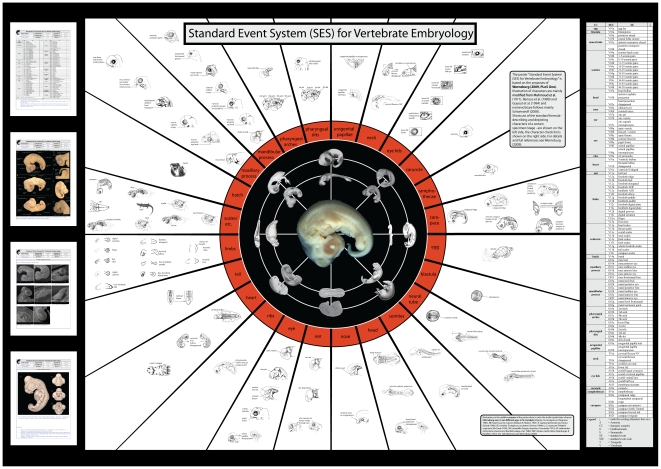
Shortcut of the supplementary SES-poster. To enable a fast survey of all characters and character complexes defined in the presented study a high resolution laboratory poster template is provided (Poster S1). The example formula (Figure 5–8) and the whole checklist of SES-characters (Figure 2–4) are also shown within.

## Discussion

### Embryology and Phylogeny

Embryos do not show a highly conserved stage with a common set of morphological features [48]. However, historically embryos of different clades showing apparent similarities were described as exhibiting a ‘phylotypic stage’ [49]. This hypothesis associated with typological thinking [50] goes back to the comparative approaches of von Baer [51] and the evolutionary interests of Haeckel [52]–[53]. Haeckel arranged different species in developmental rows showing similar stages (**Figure 1**) – on the one hand to illustrate similarities between species with a didactic approach and on the other hand to support his idea of embryonic recapitulation (*Biogenetisches Grundgesetz*) [54]. Although Haeckel recognised advanced developmental shifts between embryos of different species – he named them caenogeneses [55] - he modified and simplified several embryos, a fact that has been often criticised [see 56]–[57].

In the last few years, scientists refrained from typological thinking in comparative embryology. New methods to analyse phylogenetic patterns in comparative embryology were developed and organismic developmental biology became a new and valuable source for evolutionary research that has previously been ignored [57]–[60]. Methods to analyse such patterns are established or are under development, i.e. event pairing [5], [24]–[25], event pair cracking [6], Parsimov [7], PGi [8] and others [26]–[27].

Using all these different methods, clade supporting heterochronic shifts have been detected for gnathostomes [61], amniotes [6], [45], mammals [5], and also for snails [62] and crustaceans [63]. There are heterochronies at different levels of organogenesis and even among closely related species: neural crest [64]–[65] and cranial muscle development [60] in frogs, ossification and suture closure patterns in different tetrapod groups [66]–[69]. For these studies an initial uniformity of character definition was used – e.g. the onset of alizarin-red-staining in cleared and stained specimens [70] when studying ossification patterns. Nevertheless, studies on external and all other developmental features remain inconsistent [6], [71] or no comparable studies exist on the same organ systems.

### Advantages of a Standard Event System (SES)

#### 1. Sequence Heterochrony

Using SES, sequence heterochrony studies [i.e. 45], [60], [67]–[68], [72] can be extended easily to include more clades, species, a broader range of specimens and characters (**Figure 2** **–** [Fig pone-0005887-g003] **4**, **Table S1** **,** **S2**). When comparing SES-staged embryonic series with embryos of older studies, a problem can be identified: presently, in most cases ‘staging tables/normal stages’ are based on comparisons of more specimens than resulting ‘stages’. These ‘stages’ were created by a subjective categorical approach of the author himself or that of his reference author (**Table S3**). Drawings may represent a typified embryo of a ‘stage’ and photographs, as mainly shown in the papers, can only represent one particular specimen that may illustrate all described characters of a particular ‘stage’. Apart from somite development, no variation between specimens of one species was recorded by most authors around one ‘stage’. Hence it is possible that much information about variation and character specifications has been lost by processing these typological categorisations.

#### 2. Intra-/Interspecific Variability

The SES-formula can be used to document character sets of several specimens of one species. With regard to one reference character existing in every specimen, such as “tail bud is formed”, variation of other characters like “somite number” or development of “pharyngeal arches” can be documented for one species. Using empirical methods for analysing intraspecific variability in a phylogenetic context [29]–[30] the formulas can serve as data matrices.

#### 3. Twofold Extendibility

I provide a simple three-part SES-Code (**Figure 2** **–** [Fig pone-0005887-g003] **4**) referring to monophyletic groups, to character complexes, and to defined developmental states of these character complexes (see above). Using this scheme additional clades (i.e. Mammalia, M), character complexes (i.e. hairs, M02) and events, also for existing character complexes (i.e. upper eyelid forms, A01g) can be added. Not only external developmental features, also internal morphological, physiological, genetic or molecular characters in ontogeny can be included into the SES.

#### 4. Embryological Collections

Embryological collections are an essential source for research in comparative embryology [73]–[74]. To quickly ascertain which characters an embryo shows, the SES-formula can be used to list detailed information on a collection object, i.e. when searching for specimens having a “tail bud” [48], a “forelimb bud with AER” [75], or marsupials embryos having ossified forelimb bones in early stages [76].

#### 5. Common Language in Evo-Devo

Staging tables of so called “model organisms” like the chicken [16] are commonly used as references in Evo-Devo-research. Staging tables only represent a synopsis of several specimens, which look more or less similar. Intraspecific variability, which is generally ignored in staging tables, may lead to several communication errors. For example, the situation whereby two laboratories work on the same species: Regarding to reference paper X a specimen A is staged as stage X12 in one lab. This specimen processes a specific molecule in the mandibular arch bud. However, members of a second lab do not find this molecule in a specimen B, which is also staged as stage X12. When comparing the presented SES-Formula of these two specimens, it could be recognised that in specimen A the lower eye lid – which was not described in staging reference X – has already developed while in specimen B a lower eyelid has not yet formed. It could be thus inferred that the lower eyelid may influence the processing of the molecule in the species. When discussing molecular features of one specimen, the set of SES-characters as defined here should also be provided for clear communication.

#### 6. Staging System vs. “Staging Tables”

When describing a new series of embryos, depending on the extent of material, I suggest to describe only specimens rather than synopses of them (‘Staging Table’ concept of Harrison [15]: several similar specimens summarised to one single stage). In this way topological simplifications can be avoided and variability, crucial in intraspecific development, is not neglected. Terms as previously and typologically used such as ‘Staging Table’ [77] (**Figure 1**), ‘Normal development/stages’ [15], [20], [78] or simply ‘series of stages’ [34] should be avoided and using at least the SES based set of characters a new described series of embryos from hereon should be called a “Staging System”. Wanek et al. [79] sensibly used this term before for only two specific and clearly described character complexes: the fore- and hind limb.

### Recommendations on how to use the Standard Event System [SES]

#### A. Cataloguing in Collections

Scientific collections housing embryos simply need to provide a basic check list (**Figure 5**) of SES-characters visible in a specimen (sheet 1 of **Table S1** **or 2**). Most collections already have an online database of their catalogued objects. Linked to the listed embryo information, the filled formulas can be made available as a pdf file. Scientists describing embryos from collections may publish complete SES-formulas (**Figure 5** **–** [Fig pone-0005887-g006] [Fig pone-0005887-g007] **8**) online and connect them to the collection listings.

#### B. Documentation in Evo-Devo

When recording a discrete morphological event (e.g., describing e.g. the onset of gene X expression in limb bud development) a SES-formula could be filled out for each observed specimen. Therefore the check list (**Figure 5**) and at least the first page of illustrated documentation (**Figure 6**) should be filled out (lateral, dorsal, ventral view). More detailed illustration may be added depending on the observed region (only limbs, surrounding area like heart/liver-bulbus etc.). The laboratory poster (**Figure 9**, **Poster S1**) showing all SES-characters may be used for an exploratory survey when discussing developmental features.

#### C. Creating Staging Systems

I recommend one should describe specimens of an embryonic series instead of typological stage-synopses. Except for some r-strategy species such as the chicken [16], sea turtles [43], diverse fishes [20], frogs [80]–[81] or crocodiles [82]–[83] in general only a few embryonic specimens are available for k-strategy vertebrate species [compare **Table S3**]. To describe the external development of embryonic series I suggest the following protocol:

- Ordering: Primarily, specimens should be ordered by a comparative synopsis of the embryos' age and the development of reference organs, such as the number of somites, or as often used, the limb bud development [78]–[79]. In documenting all existing surrounding SES-characters, variability can be managed in a traceable way. The breeding temperature when known should be noted for all non-therian species (**Figure 5**).
- Numbering specimens: All specimens ordered consecutively should be numbered as specimen 1, specimen 2, specimen 3, specimen 4, specimen 5, etc.
- Coding SES-characters: The checklist (**Figure 5**) for each specimen could be presented as an appendix to the main descriptive part of the work. If an online data base is established, the checklist information should be entered there. In the main body of the text a full listing of SES-characters as observed in the specimens 1, 2, etc. should be presented.
- Illustration style: Drawings and photographs should be provided in the proposed style of the formula (lateral, dorsal, ventral view, detailed views of special characters) for each specimen if possible. Drawings/photographs need to show all SES-characters observed in a specimen (**Figure 6**). Additional illustrations are optional, such as raster-electronic-microscopy-scans (**Figure 7**). Illustrations of reference papers may be added to the formula (**Figure 8**). Figure plates should be provided in the main body of the article summarising all SES-stages to obtain an overview of species development. Authors should be aware of the biases introduced by using different methods to document a particular event and comparing different species. For example, in this study I used light microscopy to record the appearance of the apical epidermal ridge: Perhaps other imaging techniques (e.g., computer-tomography) would detect such an event earlier.
- Additional characters: Space is provided in the SES-formula to add characters that obviously are identical for only one species, such as colouration, pigmentation, or diverse scale development (Fig 2a: below). In this study I only observed a few mammalian species opposing a broad range of sauropsid species. Because of this low comparability I refrained from listing mammalian specific characters like “hairs on head (M02a)” in the SES-formula. Further studies on vertebrate embryology may provide additional character complexes and events occurring within, which could be coded in the SES-style. For actinopterygian fishes, characters like “number of dorsal fin rays up to five (AC01a)” are imaginable. In an online data base an ongoing discussion and updating of SES-characters and specimens would be possible and desirable.
- Additional specimens: In the case of species for which only a rare set of embryonic specimens exists as in the case for the echidna *Tachyglossus aculeatus* [1] or the tuatara *Sphenodon punctatus* [84] the study of new embryos may fill some gaps in understanding these series. Given a Staging System of species A with age/somite ordered specimens 1, 2, 3, 4 and 5: When finding a new embryo which is developmentally difficult to settle between specimen 2 and 3 with more similarities to specimen 2, the new specimen is to be named as “specimen 2>3”. If a further specimen is found to be settled between specimen 2 and 3 - having much more similarities to specimen 2 - it should be called “specimen 2≫3”. Although this system would be endlessly extendable, authors could decide to create a new Staging System for the specimen when having much more specimens than previously published.
- Stages vs. specimens: When having dozens or hundreds of embryos representing an embryonic series [16] it would not be practical to describe each specimen separately. In this case a synopsis has to used, as previously called normal stages, staging tables (see above). For this I propose the antiquated typological approach, but: Only specimens showing a high degree of similarity – which is measurable and documentable by the composition set of their SES-characters – should be concluded as one synoptic stage. By documenting differences in SES-composition, variability is traceable and its patterns can be calculated afterwards. When describing and illustrating SES-stages the same protocol should be followed with the same diligence as described for the specimen staging approach.

### Ontogeny and Ontology

An online database is necessary to make information of all vertebrate embryos available to the scientific community. Therein image-based as well as tabular presentations of embryological characters are necessary. It is a challenge for bioinformaticians to coordinate the enormous amount of published information on morphological, developmental and molecular data in a traceable way [85]–[87]. In morphology, for example, several scientific groups developed online databases, such as Digimorph [88], to coordinate and to provide access to the enormous amount of information available. For particular “model organisms” extensive online documentation (including embryology) is already available, such as the Edinburgh mouse atlas project, the *Xenopus*-, *C. elegans*- or *Drosophila*-project (e.g. summarised by Bio-Ontologies [89]). In addition, several web pages provide a collection of biological ontologies of a molecular and morphological kind such as Bioportal [90] or Obofoundry [91].

Commendable efforts exist to standardise anatomical nomenclature in comparative online-projects such as Phaenoscape [87], [92] for teleost fishes or the Morphological web database [93]–[94] for mammals. The study presented here also aims to set a standard reference for describing developmental anatomical features. It is intended to eventually integrate the SES to an image based internet-ontology (such as MorphDBase [95]), where information and illustrations for new species, new specimens and new SES-characters – verified by results from peer-reviewed publications – can be added individually.

## Methods

### Taxonomic sampling

I examined mostly literature data (**Table S3**) on the external development of 15 turtle species (*Apalone spinifera*, *Caretta caretta*, *Carettochelys insculpta*, *Chelonia mydas*, *Chelydra serpentina*, *Chrysemys picta*, *Dermochelys coriacea*, *Emydura subglubosa*, *Eretmochelys imbricata*, *Graptemys nigrinoda*, *Lepidochelys olivacea*, *Natator depressa*, *Testudo hermanni*, *Trachemys scripta*, *Pelodiscus sinensis*) and eight species out of the major clades of Tetrapoda: *Ambystoma mexicanum* (Lissamphibia), *Tachyglossus aculeatus* (Mammalia, Monotremata), *Didelphis virginiana* (Mammalia, Marsupialia), *Dasypus hybridus* (Mammalia, Placentalia), *Gallus gallus* (Aves), *Alligator mississippiensis* (Crocodylia), *Sphenodon puctatus* (Sphenodontida) and *Lacerta vivipara* (Squamata).

I had access to an embryonic series of the turtles *Emydura subglobosa*, the first pleurodire turtle ever observed in its external embryology [45], and one of *Graptemys nigrinoda* (Cryptodira). For *Caretta caretta*, *Chelonia mydas*, *Lepidochelys olivacea* and *Pelodiscus sinensis* (housed in the collection of Marcelo R. Sánchez-Villagra, Paläontologisches Institut und Museum der Universität Zürich) I expanded the existing information with additional specimens that were staged after the original papers (**Table S3**). The remaining tetrapod species were chosen based on the extent of described stages and the usability of the published figures.

For the echidna, *Tachyglossus aculeatus*, the staging table of Semon [1], [96]–[97] was used, which begins at a stage of 39 somite pairs and ends at a stage where hairs are visible on back and limbs. In the Hubrecht laboratory in Berlin [73]–[74] I had access to 21 embryo photographs and drawings (made by different scientists), ordered them chronologically by the number of somites, and defined 13 stages prior to the first Semon- and two stages after the last Semon-stage.

### Definition of developmental characters

All characters described in reference papers (**Table S3**) were compared and the availability of each character was scrutinised for all species. Based on this comparison, a simple, easily recognisable list of newly occurring characters during embryogenesis (organogenesis, maturation until hatching/birth) was prepared, defining 104 developmental events (**Figure 2** **–** [Fig pone-0005887-g003] **4**) comprising the following aspects: One egg, one blastula, four neural tube, eleven somite, three general head, two nose, three ear, seven eye, one rib, three heart, one tail, 14 limb, nine scale/scute/feather, one hatch, six maxillary process, eight mandibular process, five pharyngeal arch, five pharyngeal slit, two urogenital papillae, three neck, six eyelid, one egg tooth, one labial and six carapace characters. Most of the characters are generally applicable to all vertebrates.

I refrained from defining characters that, although widespread, involve much subjectivity when coding, or for which the homologisation cannot be identified (reliability) such as the shape and development of pigmentation, detailed carapace or scale differentiations – all characters important for staging pre-hatching/pre-birth embryos of a particular species. Although pigmentation and colouration is mentioned in most of the staging tables the first occurrence of pigments cells are in most cases hard to see in photographs and in embryos fixed for a long time. Other authors called an organ pigmented [41], when the organ is completely “dark”. I also refrained from listing every single somite number because there is a high variability in the formation of the mesodermal segments [98]. The presented definition of clusters, five somites each, reflects the variance of somite development in my own observation but because of the arbitrariness of the number chosen (5) is a source of subjectivity.

When defining events for the limb development either both limbs are separately or both are jointly discussed, or only the forelimb was discussed. The forelimb is described more carefully than the hind limb in some works [34] or a generally contemporary development of the features, as I experienced in observing both limbs, is visible [see also 75], [99].

If possible both body sides were observed, either in the original specimens or in the pictures. In general I did not find important differences in contralateral development. Nevertheless, if there were slight differences, both sides clearly fit to the same explicit defined event. That is especially mentionable for differing counts of somites, fitting in the same somite cluster. i.e. having 12 somites on the left body side and 13 somites on the right body side: both sides fit to the same somite cluster “11–15” (**Figure 2**: V04d).

The characters described are illustrated in **Figure 2** **–** [Fig pone-0005887-g003] **4**; most drawings are modified from Guyout et al. [40], Renous et al. [44] and Mahmoud et al. [100]. These studies present detailed and useful depictions of characters used in the staging guide I present here. Nomenclature mainly follows that of Schoenwolf [101].

## Supporting Information

Table S1 Template of a SES-formula to document developmental series and embryo specimens (in doc-format).
(0.16 MB DOC)

Table S2 Template of a SES-formula to document developmental series and embryo specimens (in pdf-format)
(0.06 MB PDF)

Table S3 Species used in this study.
(2.27 MB PDF)

Poster S1 Template for printing the illustrated standard characters in a poster format.
(20.49 MB PDF)
