# Supplementary material for: A Standard System to Study Vertebrate Embryos
Source: PLoS One. 2009 Jun 12;4(6):e5887. doi: 10.1371/journal.pone.0005887 (PMC2693928; doi:10.1371/journal.pone.0005887)
Supplement: Table S1 — Template of a SES-formula to document developmental series and embryo specimens (in doc-format). (0.16 MB DOC) [file pone.0005887.s001.doc]

| **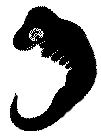**  **Standard Event System for Vertebrate Embryology** | | | | | | | | | | |
| --- | --- | --- | --- | --- | --- | --- | --- | --- | --- | --- |
| **species (group)** | | | **stage/specimen** | |  | | | **specimen/stage No.** |  | |
|  | | | **breeding temp.** | |  | | | **collection No.** |  | |
|  |  |  | **age (days)** | |  | | | **sheet No.** | 1 / | |
|  | | | | | | | | | | |
| **CC** | **SEC** | **SE** | | **↓** | **CC** | **SEC** | | **SE** | | **↓** |
| **egg** | V01a | egg lay | |  | **scales/etc.** | V13a | | head scales | |  |
| **blastula** | V02a | blastoporus | |  |  | V13b | | throat scales | |  |
| **neural tube** | V03a | primitive streak | |  |  | V13c | | eyelid scales | |  |
|  | V03b | neural folds closure | |  |  | V13d | | neck scales | |  |
|  | V03c | anterior neuropore closed | |  |  | V13e | | back scales | |  |
|  | V03d | posterior neuropore closed | |  |  | V13f | | limb scales | |  |
| **somites** | V04a | somites hard count | |  |  | V13g | | whole forelimb scales | |  |
|  | V04b | 1-5 somite pairs | |  |  | V13h | | tail scales | |  |
|  | V04c | 6-10 somite pairs | |  |  | V13i | | carapace scutes | |  |
|  | V04d | 11-15 somite pairs | |  | **hatch** | V14a | | hatch | |  |
|  | V04e | 16-20 somite pairs | |  | **maxillary process** | G01a | | max bud | |  |
|  | V04f | 21-25 somite pairs | |  |  | G01b | | max posterior eye | |  |
|  | V04g | 26-30 somite pairs | |  |  | G01c | | max midline eye | |  |
|  | V04h | 31-35 somite pairs | |  |  | G01d | | max anterior lens | |  |
|  | V04i | 36-40 somite pairs | |  |  | G01e | | max anterior eye | |  |
|  | V04j | 41-45 somite pairs | |  |  | G01f | | max frontonasal fuse | |  |
|  | V04k | 46-50 somite pairs | |  | **mandibular process** | G02a | | mand arch bud | |  |
| **head** | V05a | head bulbus | |  |  | G02b | | mand posterior eye | |  |
|  | V05b | anterior cephalic projection | |  |  | G02c | | mand posterior lens | |  |
|  | V05c | head projection disappeared | |  |  | G02d | | mand midline eye | |  |
| **nose** | V06a | olfactory pit | |  |  | G02e | | mand anterior lens | |  |
|  | V06b | external nares | |  |  | G02f | | mand anterior eye | |  |
| **ear** | V07a | otic pit | |  |  | G02g | | mand level frontonasal | |  |
|  | V07b | otic vesicle | |  |  | G02g | | mand occlusion point | |  |
|  | V07c | otic capsule inconspicuous | |  | **pharyngeal arches** | G03a | | 2nd arch | |  |
| **eye** | V08a | optic vesicle | |  |  | G03b | | 3rd arch | |  |
|  | V08b | lens vesicle | |  |  | G03c | | 4th arch | |  |
|  | V08c | optic fissure | |  |  | G03d | | 5th arch | |  |
|  | V08d | contour lens/iris | |  |  | G03e | | hyoid flap | |  |
|  | V08e | pupil forms | |  | **pharyngeal slits** | G04a | | 1st slit | |  |
|  | V08f | scleral papillae | |  |  | G04b | | 2nd slit | |  |
|  | V08g | scleral papillae inconspicuous | |  |  | G04c | | 3rd slit | |  |
| **ribs** | V09a | rib primordia | |  |  | G04d | | 4th slit | |  |
| **heart** | V10a | Ventricle bulbus | |  |  | G04e | | slits closed | |  |
|  | V10b | thoracal bulbus disappeared | |  | **urogenital papillae** | G05a | | urogenital papilla bud | |  |
|  | V10c | ventricle S-shaped | |  |  | G05b | | urogenital papilla inconspicuous | |  |
| **tail** | V11a | tail bud | |  | **neck** | T01a | | cervical flexure 90° | |  |
| **limbs** | V12a | forelimb ridge | |  |  | T01b | | cervical flexure disappeared | |  |
|  | V12b | forelimb bud | |  |  | T01c | | wrinkles on neck | |  |
|  | V12c | forelimb elongated | |  | **eye lids** | A01a | | lower lid | |  |
|  | V12d | forelimb AER | |  |  | A01b | | eyelid begun overgrow | |  |
|  | V12e | hindlimb AER | |  |  | A01c | | eyelid at scleral papillae | |  |
|  | V12f | forelimb elbow | |  |  | A01d | | eyelid ventral lens | |  |
|  | V12g | forelimb paddle | |  |  | A01e | | eyelid half eye | |  |
|  | V12h | hindlimb paddle | |  |  | A01f | | membrana nictitans | |  |
|  | V12i | forelimb digital plate | |  | **caruncle** | A02a | | caruncle | |  |
|  | V12j | hindlimb digital plate | |  | **ramphothecae** | S01a | | ramphothecae | |  |
|  | V12k | digital grooves | |  | **carapace** | S02a | | carapacial ridge | |  |
|  | V12l | digital serration | |  |  | S02b | | longitudinal carapacial ridge | |  |
|  | V12m | finger | |  |  | S02c | | carapace not anterior | |  |
|  | V12n | first claw | |  |  | S02d | | carapace clearly limited | |  |
| ***Legend:*** ↓ = mark the existing characters here as x | | | | |  | S02e | | carapace beyond tail | |  |
|  |  |  |  |  |  | S02f | | carapace irregular | |  |
| **notes** | | | | | | | | | | |
|  | | | | | | | | | | |
| **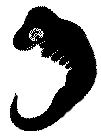**  **Standard Event System for Vertebrate Embryology** | | | | | | | | | | |
| **species (group)** | | | **stage/specimen** | |  | | | **specimen/stage No.** |  | |
|  | | | **breeding temp.** | |  | | | **collection No.** |  | |
|  |  |  | **age (days)** | |  | | | **sheet No.** | 2 / | |
|  | | | | | | | | | | |
| **drawings / photographs / references** | | | | | | | | | | |
| lateral view | | | | | | | detailed views,  related pictures  from literature | | | |
| dorsal view | | | | | | |  |  |  |  |
| ventral view | | | | | | |  |  |  |  |
| Fig. F1: | | | | | | | | | | |
| **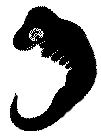**  **Standard Event System for Vertebrate Embryology** | | | | | | | | | | |
| **species (group)** | | | **stage/specimen** | |  | | | **specimen/stage No.** |  | |
|  | | | **breeding temp.** | |  | | | **collection No.** |  | |
|  |  |  | **age (days)** | |  | | | **sheet No.** | / | |
|  | | | | | | | | | | |
| **drawings / photographs / references** | | | | | | | | | | |
|  | | | | | | | | | | |
