# Supplementary material for: A Standard System to Study Vertebrate Embryos
Source: PLoS One. 2009 Jun 12;4(6):e5887. doi: 10.1371/journal.pone.0005887 (PMC2693928; doi:10.1371/journal.pone.0005887)
Supplement: Table S2 — Template of a SES-formula to document developmental series and embryo specimens (in pdf-format) (0.06 MB PDF) [file pone.0005887.s002.pdf]

Standard Event System for Vertebrate Embryology

species (group)	stage/specimen		specimen/stage No.	
	breeding temp.		collection No.	
	age (days)		sheet No.	1 /

CC	SEC	SE	↓
egg	V01a	egg lay	
blastula	V02a	blastoporus	
neural tube	V03a	primitive streak	
	V03b	neural folds closure	
	V03c	anterior neuropore closed	
	V03d	posterior neuropore closed	
somites	V04a	somites hard count	
	V04b	1-5 somite pairs	
	V04c	6-10 somite pairs	
	V04d	11-15 somite pairs	
	V04e	16-20 somite pairs	
	V04f	21-25 somite pairs	
	V04g	26-30 somite pairs	
	V04h	31-35 somite pairs	
	V04i	36-40 somite pairs	
	V04j	41-45 somite pairs	
	V04k	46-50 somite pairs	
head	V05a	head bulbus	
	V05b	anterior cephalic projection	
	V05c	head projection disappeared	
nose	V06a	olfactory pit	
	V06b	external nares	
ear	V07a	otic pit	
	V07b	otic vesicle	
	V07c	otic capsule inconspicuous	
eye	V08a	optic vesicle	
	V08b	lens vesicle	
	V08c	optic fissure	
	V08d	contour lens/iris	
	V08e	pupil forms	
	V08f	scleral papillae	
	V08g	scleral papillae inconspicuous	
ribs	V09a	rib primordia	
heart	V10a	Ventricle bulbus	
	V10b	thoracal bulbus disappeared	
	V10c	ventricle S-shaped	
tail	V11a	tail bud	
limbs	V12a	forelimb ridge	
	V12b	forelimb bud	
	V12c	forelimb elongated	
	V12d	forelimb AER	
	V12e	hindlimb AER	
	V12f	forelimb elbow	
	V12g	forelimb paddle	
	V12h	hindlimb paddle	
	V12i	forelimb digital plate	
	V12j	hindlimb digital plate	
	V12k	digital grooves	
	V12l	digital serration	
	V12m	finger	
	V12n	first claw	

Legend: ↓ = mark the existing characters here as x

CC	SEC	SE	↓
scales/etc.	V13a	head scales	
	V13b	throat scales	
	V13c	eyelid scales	
	V13d	neck scales	
	V13e	back scales	
	V13f	limb scales	
	V13g	whole forelimb scales	
	V13h	tail scales	
	V13i	carapace scutes	
hatch	V14a	hatch	
maxillary process	G01a	max bud	
	G01b	max posterior eye	
	G01c	max midline eye	
	G01d	max anterior lens	
	G01e	max anterior eye	
	G01f	max frontonasal fuse	
mandibular process	G02a	mand arch bud	
	G02b	mand posterior eye	
	G02c	mand posterior lens	
	G02d	mand midline eye	
	G02e	mand anterior lens	
	G02f	mand anterior eye	
	G02g	mand level frontonasal	
	G02g	mand occlusion point	
pharyngeal arches	G03a	2nd arch	
	G03b	3rd arch	
	G03c	4th arch	
	G03d	5th arch	
	G03e	hyoid flap	
pharyngeal slits	G04a	1st slit	
	G04b	2nd slit	
	G04c	3rd slit	
	G04d	4th slit	
	G04e	slits closed	
urogenital papillae	G05a	urogenital papilla bud	
	G05b	urogenital papilla inconspicuous	
neck	T01a	cervical flexure 90°	
	T01b	cervical flexure disappeared	
	T01c	wrinkles on neck	
eye lids	A01a	lower lid	
	A01b	eyelid begun overgrow	
	A01c	eyelid at scleral papillae	
	A01d	eyelid ventral lens	
	A01e	eyelid half eye	
	A01f	membrana nictitans	
caruncle	A02a	caruncle	
ramphothecae	S01a	ramphothecae	
carapace	S02a	carapacial ridge	
	S02b	longitudinal carapacial ridge	
	S02c	carapace not anterior	
	S02d	carapace clearly limited	
	S02e	carapace beyond tail	
	S02f	carapace irregular	

notes

Standard Event System based on Werneburg (2009, PLoS One),
"A standard system to study vertebrate embryos"

Standard Event System for Vertebrate Embryology

species (group)	stage/specimen		specimen/stage No.	
	breeding temp.		collection No.	
	age (days)		sheet No.	2 /

drawings / photographs / references	
lateral view	detailed views,<br>related pictures<br>from literature
dorsal view	
ventral view	
Fig. F1:	

Standard Event System for Vertebrate Embryology

species (group)	stage/specimen		specimen/stage No.	
	breeding temp.		collection No.	
	age (days)		sheet No.	/

drawings / photographs / references
