# Supplementary material for: A Standard System to Study Vertebrate Embryos
Source: PLoS One. 2009 Jun 12;4(6):e5887. doi: 10.1371/journal.pone.0005887 (PMC2693928; doi:10.1371/journal.pone.0005887)
Supplement: Table S3 — Species used in this study. (2.27 MB PDF) [file pone.0005887.s003.pdf]

Table S3: **Species used in this study.**

No	classification	species	Reference (main references underlined)	observed specimens	described stages (followed staging table: stages)	stages since (incl.) blastoporus [documented as pictures]
1	Caudata	*Ambystoma mexicanum*	1. Bordzilovskaya et al. (1989) (stages 1-44), 2. Nye et al. (2003) (stages 45-57)	n.d.	51 stages including "half stages" (Bordzilovskaya et al. 1989); 13 (Nye et al. 2003)	42 [42] +13 [13]
2	Mammalia, Monotremata	*Tachyglossus aculeatus*	**1. this study** (stages IW1-13, Semon-54,55)<br>2. Semon (1894a, b, c) (stages 40-53)	1. 22<br>2. n. d.	14 (this study: IW1-13, Semon 54), 14 (Semon 1894c: 40-53)	13 [13] + 14 [14]
3	Mammalia, Theria, Marsupialia	*Didelphis virginiana*	McCrandy (1938)	n.d.	35 (McCrandy 1938: 1-35)	20 [20]
4	Mammalia Theria, Placentalia	*Dasypus hybridus (=Tatusia hybrida)*	Fernandez (1915)	206+	35 (Fernandez 1915: 1-35)	24 [21]
5	Archosauria, Aves	*Gallus gallus*	Hamburger & Hamilton (1951)	996+	45 (Hamburger & Hamilton 1951: 1-45)	44 [44]
6	Archosauria, Crocodylia	*Alligator mississippiensis*	1. Ferguson (1985)<br>2. Voeltzkow (1899)	1. 1500<br>2. n.d.	4 early stages (after Voeltzkow 1899), 28 (Ferguson 1985: 1-28)	32 [32]
7	Lepidosauria, Sphenodontida	*Sphenodon puctatus*	1. Dendy (1899)<br>2. Moffat (1985)	1. 90<br>2. n.d.	16 (Dendy 1899: C-S)	16 [15]
8	Lepidosauria, Squamata	*Lacerta vivipara*	1. Dufaure & Hubert (1961)<br>2. Hubert (1985)<br>3. Moffat (1985)	1. 350<br>2. n.d.<br>3. n.d.	40 (Dufaure & Hubert 1961: 1-40)	36 [36]
9	Testudines, Cryptodira, Chelonioidea	*Caretta caretta*	1. Billett et al. (1992)<br>2. Miller (1985)<br>3. **this study**	1. n.d.<br>2. 1303<br>3. 5	31 (Miller 1985: 1-31)	22 [20]
10	Testudines, Cryptodira, Chelonioidea	*Chelonia mydas*	1. Miller (1985)<br>2. Parker (1880)<br>3. **this study**	1. 723<br>2. 26+<br>3. 18	31 (Miller 1985:1-31)	22 [19]
11	Testudines, Cryptodira, Chelonioidea	*Dermochelys coriacea*	Renous et al. (1989)	97+	31 (Miller 1985: 1-31)	22 [22]
12	Testudines, Cryptodira, Chelonioidea	*Eretmochelys imbricata*	Miller (1985)	567	31 (Miller 1985: 1-31)	22 [12]
13	Testudines, Cryptodira, Chelonioidea	*Lepidochelys olivacea*	1. Crastz (1982)<br>2. **this study**	1. 210<br>2. 28	31 (Crastz 1982: 1-31)	31 [28]
14	Testudines, Cryptodira, Chelonioidea	*Natator depressa*	Miller (1985)	375	31 (Miller 1985:1-31)	22 [10]
15	Testudines, Cryptodira, Chelydridae	*Chelydra serpentina*	**Yntema (1968)**	Not noted	27 (Yntema 1968:0-26)	27 [27]
16	Testudines, Cryptodira, Emydidae	*Chrysemys picta*	Mahmoud et al. (1973)	446	23 (Mahmoud et al. 1973: 1-23)	23 [23]
17	Testudines, Cryptodira, Emydidae	*Graptemys nigrinoda*	**this study**	35	14 (Yntema 1968: 12-15, 17-26)	14 [14]
18	Testudines, Cryptodira, Emydidae	*Trachemys scripta*	Greenbaum (2002)	104	15 (Yntema 1968: 12-26)	15 [15]
19	Testudines, Cryptodira, Testudinidae	*Testudo hermanni*	Guyot et al. (1994)	161	27 (Yntema 1968: 0/1-26)	27 [27]
20	Testudines, Cryptodira, Trionychia	*Apalone spinifera*	Greenbaum & Carr (2002)	112	15 (Yntema 1968: 12-26)	15 [14]
21	Testudines, Cryptodira, Trionychia	*Carettochelys insculpta*	Beggs et al. (2000)	54	15 (Yntema 1968: 12-26)	15 [15]
22	Testudines, Cryptodira, Trionychia	*Pelodiscus sinensis*	1. Tokita & Kuratani (2001)<br>2. **this study**	1. 67<br>2. 6	23 (Tokita & Kuratani 2001: 5-27)	23 [23]
23	Testudines, Pleurodira	*Emydura subglubosa*	**this study**	18	14 (Yntema 1968: 12-15, 17-26)	14 [14]

References to Table S3:

Beggs K, Young J, Georges A, West P (2000) Ageing the eggs and embryos of the pig-nosed turtle, *Carettochelys insculpta* (Chelonia: Carettochelydidae), from northern Australia. Canadian Journal of Zoology 78: 373-392.

Bordzilovskaya NP, Dettlaff TA, Huhon ST, Malacinski GM (1989) Developmental-stage series of Axolotl embryos. In: Armstrong JB, Malacinski GM, editors. Developmental biology of the Axolotl. New York, Oxford: Oxford University Press. pp. 201-219.

Crastz F (1982) Embryological stages of the marine turtle *Lepidochelys olivacea*. Rev Biol Trop 30: 113-120.

Dendy A (1899) Outlines of the Development of the Tuatara, *Sphenodon (Hatteria) punctatus*. Quarterly Journal of Microscopical Science s2-42: 1-87.

Dufaure JP, Hubert J (1961) Table de développement du lézard vivipara: *Lacerta* (*Zootoca*) *vivipara*. Archives D'Anatomie Microscopique et de Morphologie Expérimentale 50: 307-327.

Ferguson MWJ (1985) Reproductive biology and embryology of the crocodilians. In: Gans C, Billet F, Maderson PFA, editors. Biology of the Reptilia Volume 14 - Development A. New York: John Wiley & Sons. pp. 329-491.

Fernandez M (1915) Die Entwicklung der Mulita - La embriología de la Mulita (*Tatusia hybrida* Desm.). Revista del Museo de la Plata 21: 519.

Greenbaum E (2002) A standardized series of embryonic stages for the emydid turtle *Trachemys scripta*. Canadian Journal of Zoology 80: 1350-1370.

Greenbaum E, Carr JL (2002) Staging criteria for embryos of the spiny softshell turtle, *Apalone spinifera* (Testudines: Trionychidae). Journal of Morphology 254: 272-291.

Guyot G, Pieau C, Renous S (1994) Développement embryonnaire d'une tortue terrestre, la tortue d'Hermann, *Testudo hermanni* Gmelin, 1789. Annales des Sciences Naturelles Zoologie Paris 15: 115-137.

Hamburger V, Hamilton HL (1951) A series of normal stages in the development of the chick embryo. Journal of Morphology 88: 49-92.

Hubert J (1985) Embryology of the Squamata. In: Gans C, Billet F, Maderson PFA, editors. Biology of the Reptilia Volume 15 - Development B. New York: John Wiley & Sons. pp. 1-34.

Mahmoud IY, Hess GL, Klicka J (1973) Normal Embryonic Stages of the Western Painted Turtle, *Chrysemys picta bellii*. J Morph 141: 268-280.

McCrady E, Jr. (1938) The embryology of the opossum. The American Anatomical Memoirs 16: 225.

Miller JD (1985) Embryology of marine turtles. In: Gans C, Billet F, Maderson PFA, editors. Biology of the Reptilia Volume 14 - Development A. New York: John Wiley & Sons. pp. 269-328.

Moffat LA (1985) Embryonic development and apects of reproductive biology in the tuatara, *Sphenodon punctatus*.

Nye HLD, Cameron JA, Chernoff E-AG, Stocum L (2003) Extending the table of stages of normal development of Axolotl: Limb development. Developmental Dynamics 226: 555-560.

Parker WK (1880) Report on the development of the green turtle (*Chelone viridis*, Schneid.). Green: London Longmans. 1-57 p.

Renous S, Rimblot-Baly F, Fretey J, Pieau C (1989) Caractéristique du développement embryonnaire de la tortue luth, *Dermochelys coriacea* (Vandelli, 1761). Annales des Sciences Naturelles Zoologie Paris 10: 197-229.

Schoenwolf GC (2008) Altas of descriptive embryology. San Francisco: Pearson Education. 125-195 p.

Semon R (1894a) Beobachtungen über die Lebensweise und Fortpflanzung der Monotremen nebst Notizen über ihre Körpertemperatur. Denkschriften der Medicinisch-Naturwissenschaftlichen Gesellschaft zu Jena 5: 3-15.

Semon R (1894b) Die Embryonalhüllen der Monotremen und Marsupialier. Denkschriften der Medicinisch-Naturwissenschaftlichen Gesellschaft zu Jena 5: 19-58.

Semon R (1894c) Zur Entwickelungsgeschichte der Monotremen. Denkschriften der Medicinisch-Naturwissenschaftlichen Gesellschaft zu Jena 5: 61-74.

Tokita M, Kuratani S (2001) Normal embryonic stages of the Chinese softshelled turtle *Pelodiscus sinensis* (Trionychidae). Zoological Science 18: 705-715.

Voeltzkow A (1899) Beiträge zur Entwicklungsgeschichte der Reptilien. I. Biologie und Entwicklung der äußeren Körperform von *Crocodilus madagascariensis*. Abhandlungen der Senckenbergischen Naturforschenden Gesellschaft 26: 1-150.

Yntema CL (1968) A series of stages in the embryonic development of *Chelydra serpentina*. Journal of Morphology 125: 219-251.
